# 1185. Taking care of migrants: The approach to eosinophilia

**DOI:** 10.1093/ofid/ofac492.1020

**Published:** 2022-12-15

**Authors:** Michael Kladias, Cesar G Berto Moreano, Sumaita Mahmood, Lianna Friedman, Christina Coyle

**Affiliations:** NYC Health and Hospitals/Jacobi, New York, New York; Jacobi Medical Center - Albert Einstein College of Medicine, Boston, Massachusetts; Albert Einstein College of Medicine, New York, New York; Albert Einstein College of Medicine, New York, New York; Jacobi Medical Center - Albert Einstein College of Medicine, Boston, Massachusetts

## Abstract

**Background:**

Eosinophilia in immigrant patients is a common finding in the outpatient setting. While screening guidelines for refugee populations exist, there are no guidelines to the approach to eosinophilia in immigrants. Etiologic diagnosis and laboratory investigation in migrant patients requires attention to the epidemiological risk factors. Serodiagnosis is an important tool in the diagnosis of chronic parasitic diseases affecting migrant patients.

**Methods:**

We describe the demographic, laboratory and diagnosis characteristics of migrant patients with eosinophilia (absolute eosinophil count ≥ 500 cells/mm3) who were referred to the Tropical Medicine clinic of Jacobi Medical Center, New York.

**Results:**

A total of 104 cases of eosinophilia were seen. Most individuals were female (54.8%) and born in The Caribbean (27.9%), followed by Asia (23.1%) and Central America (15.4%). The mean age of the patients was 50.7 ± 14.4 years and the median time from migration was 18 years [IQR 6-28 years]. The mean of the peak of eosinophils in the past 3 months was 1300 ± 600 cel/mm3. Serology for Strongyloides was positive in 24 patients (23.1%). The proportion of seroprevalence for Strongyloides by country was higher in Central America (7 patients; 43.8%), Africa (3 patients; 37.5%) and The Caribbean (8 patients; 27.6%). Patients with bathroom and plumbing outdoors were more likely to be seropositive for Strongyloides (p=0.04). Chagas serology was positive in 6 out of 10 patients tested and Schistosoma antibodies were positive in 3 out of 8 patients tested. Of note, both results were only tested in patients with epidemiological risk factors.

**Conclusion:**

Strongyloidiasis was common in our migrant population. Individuals without water supply and bathroom inside the house were more likely to have a positive serology. Many of these patients can develop disseminated infection if receiving corticosteroids. Screening in at-risk asymptomatic patients allows to identify and treat this latent disease and avoid potential morbidity and mortality. Patients were also screened for other latent parasitic infections based on epidemiology risk factors that do not result in eosinophilia such as Chagas disease and schistosomiasis. Further studies are needed to help inform guidelines on screening immigrant populations.

**Disclosures:**

**All Authors**: No reported disclosures.

